# Applying a nutrition security lens to the Dietary Guidelines for Americans to address metabolic health

**DOI:** 10.3389/fnut.2023.1141859

**Published:** 2023-04-21

**Authors:** Jeff S. Volek, Jonathan Clinthorne, William S. Yancy Jr

**Affiliations:** ^1^Department of Human Sciences, The Ohio State University, Columbus, OH, United States; ^2^The Simply Good Foods Company, Denver, CO, United States; ^3^Department of Medicine, Duke University School of Medicine, Durham, NC, United States

**Keywords:** nutrition security, metabolic health, low-carbohydrate diet, dietary guidance, dietary patterns

## Abstract

Nutrition security - access to food that promotes well-being and prevents or treats disease, particularly among racial and ethnic minority populations, lower income populations, and rural and remote populations - is a national priority. Leading causes of death and disability in America, such as heart disease, stroke, cancer, and diabetes that disproportionately affect minorities are linked to preventable diet-related risk factors. Mounting evidence indicates that adherence to a lower-carbohydrate dietary pattern is associated with weight maintenance, improved blood glucose and insulin control, lower blood pressure, reduced markers of inflammation, and a more favorable lipid profile. Additionally, prior concerns regarding the higher fat and cholesterol content of this dietary pattern are less founded in modern research. The inclusion of a lower-carbohydrate option that meets all essential nutrient requirements aligns with the contemporary movement toward more flexibility and precision nutrition. Most important, a lower-carbohydrate option positions the Dietary Guidelines for Americans to more accurately reflect the current scientific evidence and more effectively address the metabolic health of the nation. Further, it has the potential to improve nutrition security by addressing metabolic diseases that disproportionately affect people from historically marginalized racial, ethnic, socioeconomic, and cultural backgrounds. Given that most American adults are living with at least one diet-related chronic metabolic disease, updating the Dietary Guidelines for Americans to recognize and reflect the poor health status of the general population is prudent and urgent.

## Introduction

The USDA defines nutrition security as, “consistent access, availability, and affordability of foods and beverages that promote well-being, prevent disease, and if needed, treat disease, particularly among racial/ethnic minority populations, lower income populations, and rural and remote populations including Tribal communities and insular areas (1).” One avenue to achieving nutrition security is to shift current nutrition guidance from a broad focus on meeting recommendations for adequate calories, nutrients, or foods to a more precise approach that effectively addresses the most prevalent diet-related challenges with which Americans currently live (2, 3).

Several of the leading causes of death and disability in the U.S., such as heart disease, stroke, cancer, and diabetes are associated with diet-related risk factors, including obesity, hypertension and dyslipidemia (3). A cross-sectional study of U.S. adults conducted from 1999–2018 found that food insecurity increased over time among patients with cardiovascular disease (CVD) and cardiometabolic risk factors, and was more frequent among those with CVD (4). In this study, disparities in food insecurity persisted across racial and ethnic groups (4). Another study found that CVD, cancer, and diabetes accounted for half of annual deaths in the U.S., with people living in southern states, men, and African Americans having disproportionately higher mortality rates than people living in other regions, women, and other races, respectively (5). Within population subgroups in 2017–2018, prevalence of optimal metabolic health was 50% lower in adults with lower education compared with adults with higher education, and 38% lower in Mexican Americans compared with non-Hispanic White adults, indicating a need for national public health interventions aimed at improving cardiometabolic health equity (6).

The cost of poor cardiometabolic health in America is staggering. In the last 50 years, federal health care spending has risen from 5 to 28% of the federal budget, with U.S. business (inflation-adjusted) spending on health care increasing from $79 billion to $1,180 billion (7). According to data from the Government Accountability Office (GAO), in 2018, treating diet-related diseases accounted for more than half of total federal healthcare spending (5). Furthermore, the American Diabetes Association (ADA) reported that having a diet-related disease drove up individual healthcare costs, with people with diagnosed diabetes incurring medical expenditures that were over twice what the medical expenditures would have been if the person did not have diabetes (8).

The 2022 White House National Strategy on Hunger, Nutrition, and Health stated that the Dietary Guidelines Advisory Committee (DGAC) will review all the Dietary Guidelines for Americans’ (DGA) scientific questions with a health equity lens to ensure that the 2025–2030 DGA is inclusive of people from diverse racial, ethnic, socioeconomic, and cultural backgrounds (9). The DGAC will also explore whether additional examples of healthy dietary patterns should be developed and proposed based on population norms, preferences, and needs of the diverse individuals and cultural foodways within the U.S. population (9). Given that a majority of Americans are currently living with at least one diet-related chronic metabolic disease (10) updating the DGA to recognize and reflect the poor health status of the general population, and the even more dire health status of historically marginalized populations, is prudent at this time. This shift can be facilitated by expanding the current three USDA Food Patterns included in the DGA – Healthy U.S. Style Pattern, Healthy Vegetarian Pattern, and Healthy Mediterranean-Style Pattern – to demonstrate how they can be followed as part of a lower carbohydrate lifestyle that can improve the health of Americans.

### Healthy low-carbohydrate eating patterns reduce risk factors related to chronic diet-related diseases

Whereas there is no universally accepted definition for low-carbohydrate diets, it is preferred to define them by their absolute content in grams since variation in caloric intake influences the percent of calories derived from any macronutrient (11). The current Recommended Daily Allowance (RDA) for carbohydrate is 130 grams per day, thus we suggest low-carbohydrate diets be defined as those consisting of fewer than 130 grams per day or 26% or less of total daily calories (assuming a diet of 2,000 kcal/day). Current USDA Food Patterns included in the DGA encourage consumption of closer to 300 grams of carbohydrate per day (assuming a diet of 2,000 kcal/day). Ketogenic diets, a subset of low-carbohydrate diets, typically consist of less than 50 grams of carbohydrate per day with adequate protein and varying amounts of fat (11). As reported in multiple systemic reviews and meta-analyses (12, 13) reducing carbohydrate intake to less than or equal to 130 g/day, or 26% or less of total daily calories, is associated with weight management (14–16), improved blood glucose and insulin control (17, 18), lowered blood pressure (14, 15), decreased markers of inflammation (19), a more favorable lipid profile (14, 20), and reduced medication requirements (21), despite associated increases in dietary fat. Low-carbohydrate dietary patterns of up to 130 g/d of total carbohydrate align with the RDA for Americans at every life stage, except for pregnant and lactating women (22), while improving risk factors for diet-related chronic diseases (14, 15).

African Americans and Hispanic Americans are disproportionately affected by overweight and obesity in the U.S.; combined, these populations make up more than 75% of overweight and obese Americans (23). A systematic review and meta-analysis of thirty-seven studies with varying degrees of carbohydrate restriction classified as moderate-low carbohydrate diets of less than 45 to 40% of energy, low-carbohydrate diets of less than 40 to 30% of energy, and very-low carbohydrate diets of less than 30% energy, found that all three classifications of low carbohydrate diets resulted in significant weight loss compared to moderate-carbohydrate diets of 45–55% energy (15). Weight loss was significantly more pronounced as participants further restricted carbohydrate intake and was more prominent among participants who were overweight or obese at baseline (15). The results of this meta-analysis highlighted the potential for low-carbohydrate dietary patterns in helping today’s predominantly overweight and obese U.S. population manage weight and are a promising solution to race and ethnicity-associated weight disparities. With U.S. obesity prevalence being 41.9% as of 2017, disproportionately affecting lower socioeconomic and historically marginalized racial and ethnic groups (e.g., the prevalence of obesity in non-Hispanic black women in 2017 was 57%), and a primary driver of chronic diseases such as heart disease, stroke, type 2 diabetes, certain types of cancer (24), NAFLD and NASH (25), dietary strategies that have been demonstrated as effective means for managing weight are necessary at this time.

Carbohydrate restriction (26) and weight control can help reduce the risk for type 2 diabetes (27). It is estimated that nearly 50% of American adults are living with prediabetes or diabetes (28). In 2018, African Americans died from diabetes at a rate of 1.2 times higher than American Indians and Alaska Natives, 1.9 times higher than White people, and 2.3 times higher than Asians and Pacific Islanders (5). Further, between 2011 and 2014, compared with persons with high income, the relative percentage increase in diabetes prevalence was 40, 74.1, and 100.4% for those classified as middle income, near-poor, and poor, respectively (29). The ADA, the leading public health authority on diabetes in the U.S., recognizes a low-carbohydrate dietary pattern as a viable and preferable way of managing blood glucose for those at risk for developing diabetes, and concluded in their 2023 Standards of Care that, “for people with type 2 diabetes, low-carbohydrate and very low-carbohydrate eating patterns in particular have been found to reduce A1C and the need for antihyperglycemic medications (27).”

Another diet-related chronic condition affecting Americans is hypertension, classified as blood pressure higher than 130/80 mm Hg. Nearly half of adults in the U.S. (49.6%) have hypertension or are taking medication for hypertension (30). Elevated blood pressure increases risk for heart disease, heart attack, and stroke, and in 2020, more than half a million deaths in the U.S. were attributable to hypertension (31). High blood pressure is more common in African American adults (56%) compared to all other ethnic groups (30). A systematic review and meta-analysis of twenty-nine studies found that moderate to low-carbohydrate diets, between 40–45% of energy, resulted in significant reductions in blood pressure (15). Even better results have been demonstrated with lower carbohydrate intakes (32, 33). Strategies to reduce risk for cardiovascular-related diseases are warranted, and mounting evidence indicates low and moderate carbohydrate restriction is associated with lowering blood pressure in adults (14, 26).

Low-carbohydrate dietary patterns have also been demonstrated to improve lipoprotein profiles (15), thereby reducing risk for CVD. Significant improvements in triglyceride levels among healthy people (14, 16) or the general public-those with at least one chronic disease - (20, 26, 34, 35) have been consistent across both observational and controlled studies when people follow low carbohydrate dietary patterns. Further, it’s been demonstrated that as carbohydrate intake is decreased, regardless of the LDL-cholesterol response, the distribution of LDL-lipoproteins consistently shifts to a less atherogenic profile (19, 36).

NAFLD is the most common cause of chronic liver disease in western countries (37) and disproportionately affects Hispanic Americans (25). Ketogenic diets have been demonstrated to promote metabolic benefits in people with NAFLD (37, 38). Effects have been attributed to improved liver fat metabolism and beneficial shifts in gut microbial composition (37). The results of these studies indicate dietary patterns at or below130 g/d of carbohydrate help reduce chronic-disease risk among Americans. This is of importance when considering that the majority of adults in the U.S. are living with at least one diet-related chronic disease and the disproportionate rates of diet-related metabolic diseases within racial/ethnic minorities, lower-income, and rural populations.

### There is an opportunity to include low-carbohydrate options in the DGA dietary patterns

Diabetes Canada acknowledged that low-or very-low carbohydrate diets can be considered healthy eating patterns for individuals living with diabetes for weight loss, improved glycemic control, and/or to reduce the need for antihyperglycemic therapies, while a similar conclusion was reached in a scientific statement from the American Heart Association (39, 40). Data from randomized clinical trials indicate low-carbohydrate versions of currently recommended dietary patterns are safe and effective in reducing risk factors associated with chronic diseases. In a randomized intervention among moderately overweight, mostly male, middle-aged participants, it was demonstrated that a Mediterranean-style low-carbohydrate dietary pattern that initially reduced carbohydrate intake to less than 40 g/d, and then gradually increased to 70 g/d, effectively reduced intrapericardial-fat, the accumulation of which is associated with increased risk for CVD, more than an isocaloric standard low-fat diet that limited total fat intake to 30% of total calories (41). Another trial demonstrated that compared with the traditional Dietary Approaches to Stop Hypertension (DASH) diet, a modified version that was lower in carbohydrate and higher in fat lowered blood pressure to the same extent as the traditional DASH diet, and reduced plasma triglyceride and VLDL concentrations without significantly increasing LDL cholesterol (34). In people with type 2 diabetes, low-carbohydrate and low-saturated fat diets that contained 14% of energy as carbohydrate, 28% as protein, and 58% as fat (<10% saturated) resulted in weight loss, improvements in blood lipid profile and glucose stability, and reduced requirements for diabetes medications (42). These are just a small sampling of the extensive peer-reviewed published studies demonstrating that low-carbohydrate eating patterns are safe and highly effective at promoting weight loss and improving metabolic health (11). The results of these clinical investigations demonstrated that low-carbohydrate versions of DGA-recommended dietary patterns are an acceptable, if not preferred, means of managing chronic disease.

Other studies have also demonstrated that low-carbohydrate diets can fit into a variety of dietary preferences. A calorie-restricted, plant-based, low-carbohydrate dietary pattern consisting of 26% of total calories from carbohydrates demonstrated weight loss, favorable changes to blood lipid profiles, and reductions in systolic and diastolic blood pressure when compared with a conventional low-fat diet (43). In a three-year observational study conducted among Japanese outpatients with type 2 diabetes, a moderately low-carbohydrate diet resulted in improved blood lipids and glucose control (44).

Table 1 shows the current DGA Healthy U.S.-Style Dietary Pattern for Ages 2 and Older (22), with daily or weekly amounts from food groups, subgroups, and components alongside a proposed Lower Carbohydrate, Healthy U.S. Dietary Pattern. The proposed Lower-Carbohydrate Pattern maintains the 1,800-kilocalorie level of the eating pattern, with a decrease from six to one ounce equivalent per day from grains and 3 to 1.5 cup equivalents per day from dairy; and an increase from 2.5 to 5 cup equivalents per day from vegetables, five to twelve-ounce equivalents per day from protein foods, and twenty-four to forty-two grams per day from oils, with a limit on calories for other uses for oils set at one-hundred-forty kilocalories per day. This lower carbohydrate dietary pattern provides adequate fiber, potassium, calcium and vitamin D while reducing carbohydrates to 126 g (25% of calories), compared to 289 g (53% of calories) in the existing Healthy US Style Dietary Pattern. The proposed Lower-Carbohydrate Pattern could help Americans achieve a lower-carbohydrate lifestyle that fits within the RDA for carbohydrate, fits into the USDA Thrifty Food Plan, and is associated with weight management (14–16), improved blood glucose and insulin control (17, 18), lowered blood pressure (14, 15), and a more favorable lipid profile (14, 20).

**Table 1:** Healthy U.S.-Style Dietary Pattern for Ages 2 and Older, With Daily or Weekly Amounts From Food Groups, Subgroups, and Components (meets Thrifty Food Plan*).

	Healthy U.S.	Lower Carbohydrate, Healthy U.S.
**CALORIE LEVEL OF PATTERN**	**1,800**	**1,800**
**FOOD GROUP OR SUBGROUP**	**Daily Amount of Food From Each Group** (Vegetable and protein foods subgroup amounts are per week.)	
**Vegetables (cup eq/day)**	**2 ½**	**5** **
	Vegetable Subgroups in Weekly Amounts	
Dark-Green Vegetables (cup eq/wk)	1 ½	10 ½
Red and Orange vegetables (cup eq/wk)	5 ½	15 ¾
Beans, Peas, Lentils (cup eq/wk)	1 ½	3 ½
Starchy Vegetables (cup eq/wk)	5	1 ¾
Other vegetables (cup eq/wk)	4	3 ½
**Fruit (cup eq/day)**	**1 ½**	**½** **
**Grains (ounce eq/day)**	**6**	**1** **
Whole Grains (ounce eq/day)	3	1
Refined Grains (ounce eq/day)	3	0
**Dairy (cup eq/day)**	**3**	**½** **
**Protein Foods (ounce eq/day)**	**5**	**12** **
	Protein Foods Subgroups in Weekly Amounts	
Meats, Poultry, Eggs (ounce eq/wk)	23	34
Seafood (ounce eq/wk)	8	43
Nuts, Seeds, Soy products (ounce eq/wk)	4	7
**Oils (grams/day)**	**24**	**42** **
**Limit on Calories for Other Uses (kcal/day)**	**140**	–
Limit on Calories for Other uses (%/day)	6%	–

* Thrifty Food Plan: In October 2021, $58.32/week for female 20–50 years old in 1-person household. In November 2021, cost for 1-day menu $6.95 using state of Colorado pricing.

** Rounded down to the nearest ½ cup or ounce eq. Bold values are totals for each Dietary Guidelines for Americans food group; non-bold values are for food sub-groups.

## Discussion

The Government Accountability Office, tasked with providing Congress, the heads of executive agencies, and the public with timely, fact-based, non-partisan information that can be used to improve government and save taxpayers billions of dollars, published a report to Congressional requesters in 2021 that reviewed two-hundred efforts to improve diet quality and reduce the risk of chronic diseases from twenty-one government agencies and stated, “The increase in certain diet-related conditions over time indicates further potential threats to Americans’ health (5).” The USDA and HHS have an opportunity to recognize this public health crisis as they update the DGA and can provide options to help improve the health of an overwhelmingly sick population. The inclusion of a lower-carbohydrate option that aligns with the current scientific evidence and fits within existing dietary patterns will help the DGA more effectively target the spiraling crisis of poor metabolic health in the nation. Further, it has the potential to enhance nutrition security to address the disproportionately high prevalence of impaired metabolic health in racial/ethnic minorities, lower-income populations, and rural and remote groups who are experiencing an outsized burden of nutrition-related chronic diseases. As was stated in the White House National Strategy on Hunger, Nutrition, and Health, “The consequences of food insecurity and diet-related diseases are significant, far reaching, and disproportionately impact historically underserved communities. Yet, food insecurity and diet-related diseases are largely preventable, if we follow the best science and prioritize the health of the nation (9).”

## Data availability statement

The original contributions presented in the study are included in the article/supplementary material, further inquiries can be directed to the corresponding author.

## Author contributions

All authors listed have made a substantial, direct, and intellectual contribution to the work and approved it for publication.

## Funding

Funding support for this publication was provided by an unrestricted grant from Simply Good Foods USA, Inc.

## Conflict of interest

JC is an employee and shareholder of Simply Good Foods USA, Inc. Simply Good Foods owns Atkins® and Quest Nutrition®, brands that sell low-carbohydrate food products. JV is a co-founder and has equity in Virta Health; serves as a scientific advisor for Simply Good Foods and receives royalties from books discussing the science of low-carbohydrate eating patterns.

The authors declare that this study received funding from Simply Good Foods USA, Inc. The funder had the following involvement in the study: helped write the article and design and analyze the dietary pattern modelling.

## Publisher’s note

All claims expressed in this article are solely those of the authors and do not necessarily represent those of their affiliated organizations, or those of the publisher, the editors and the reviewers. Any product that may be evaluated in this article, or claim that may be made by its manufacturer, is not guaranteed or endorsed by the publisher.
